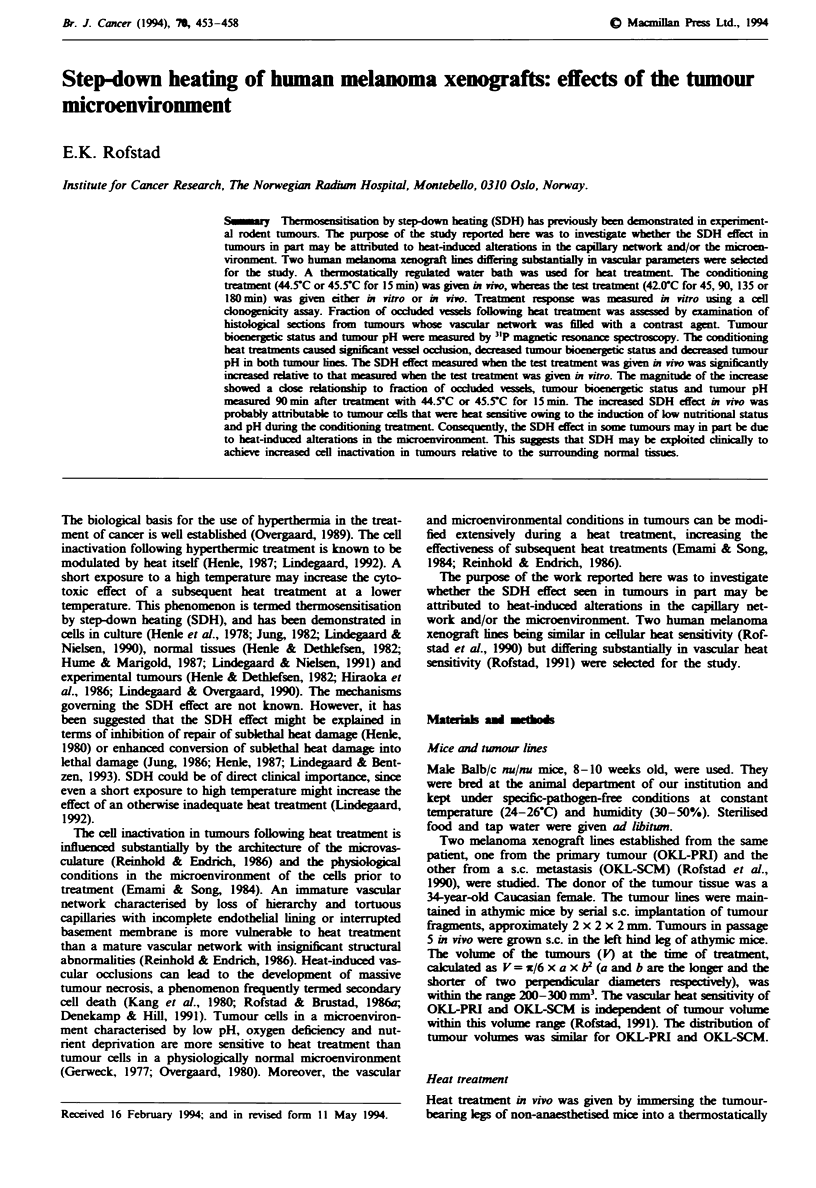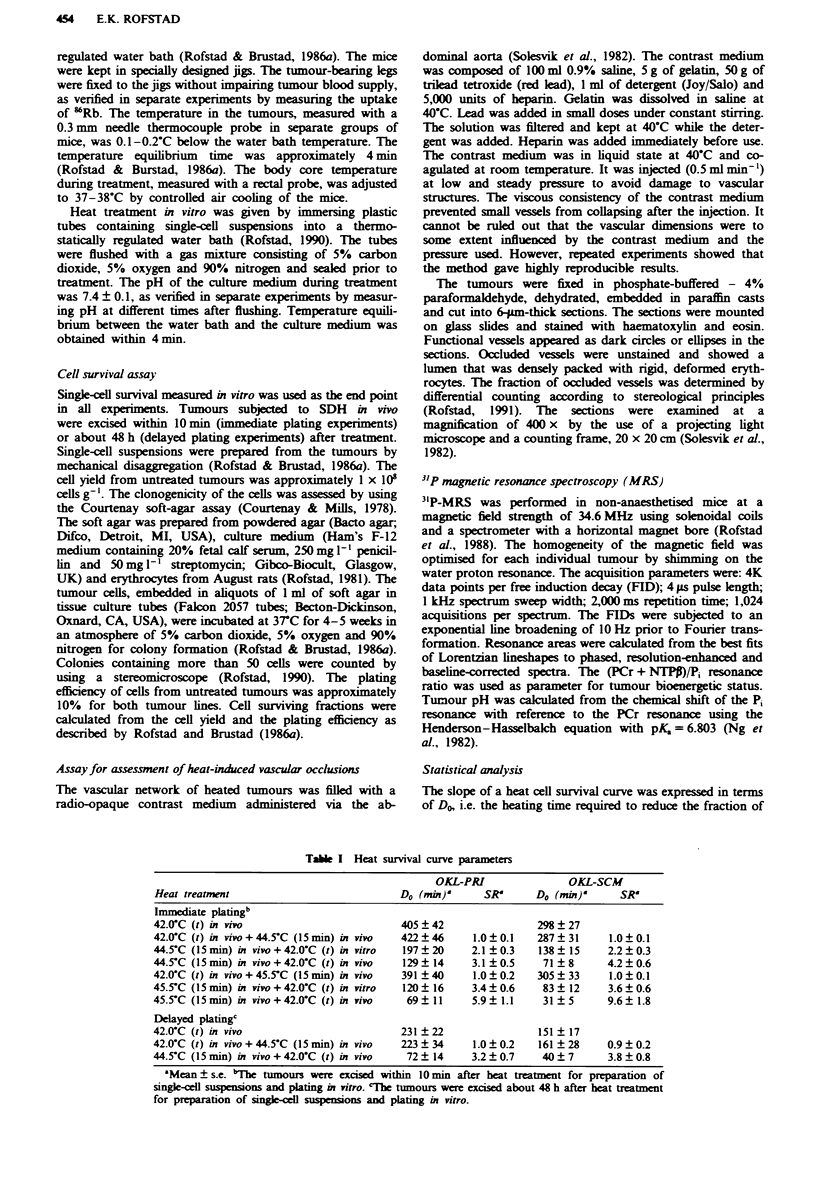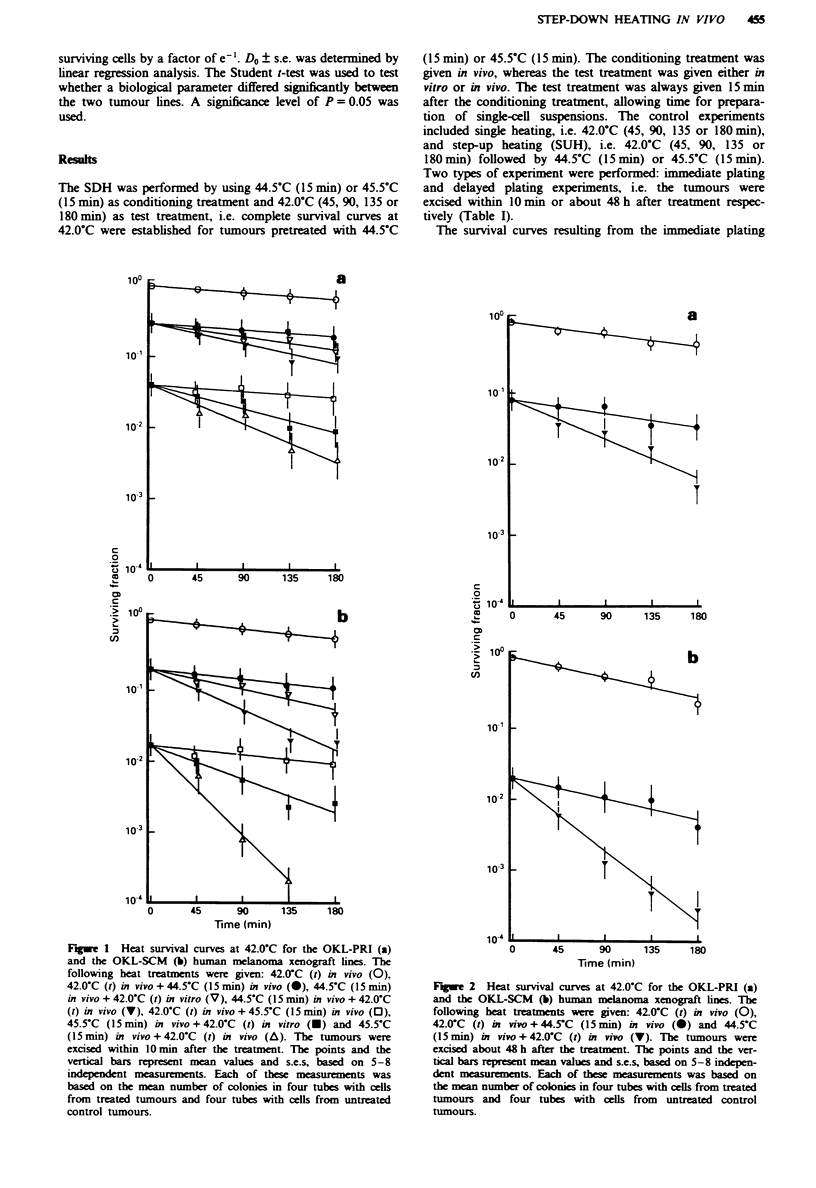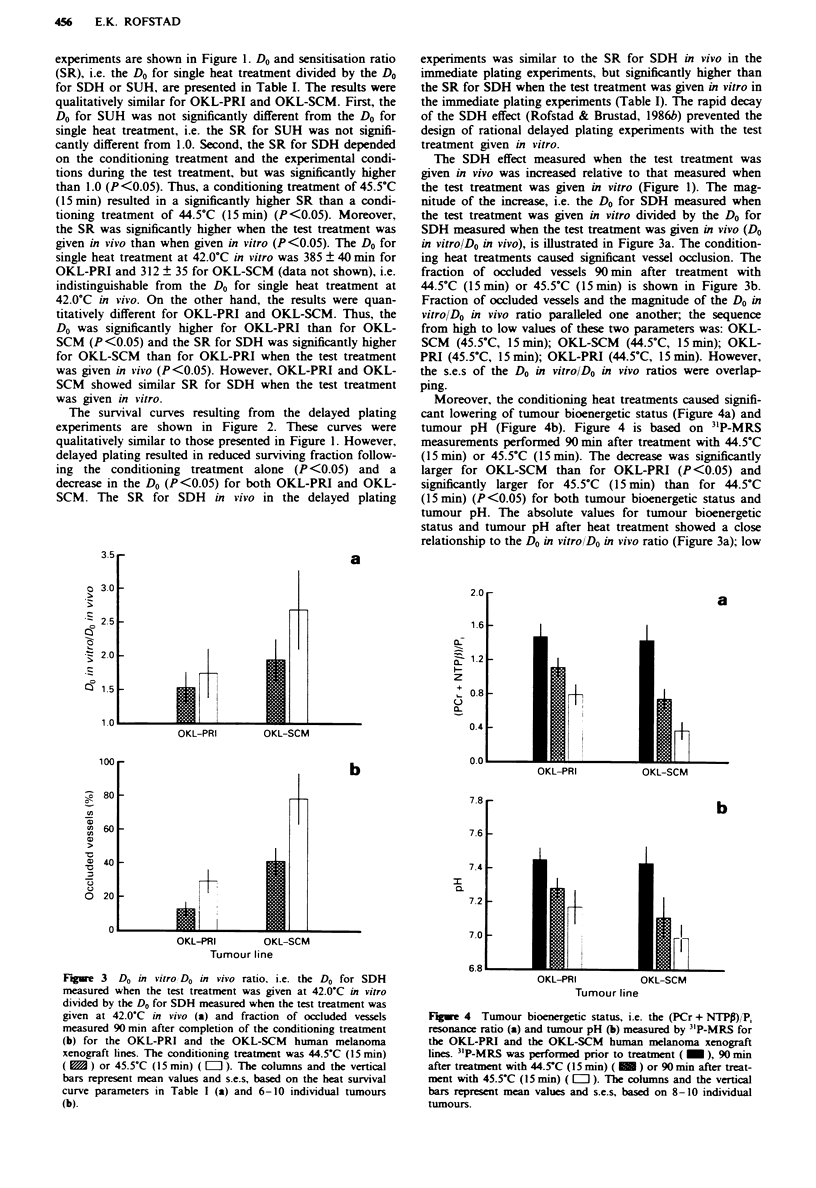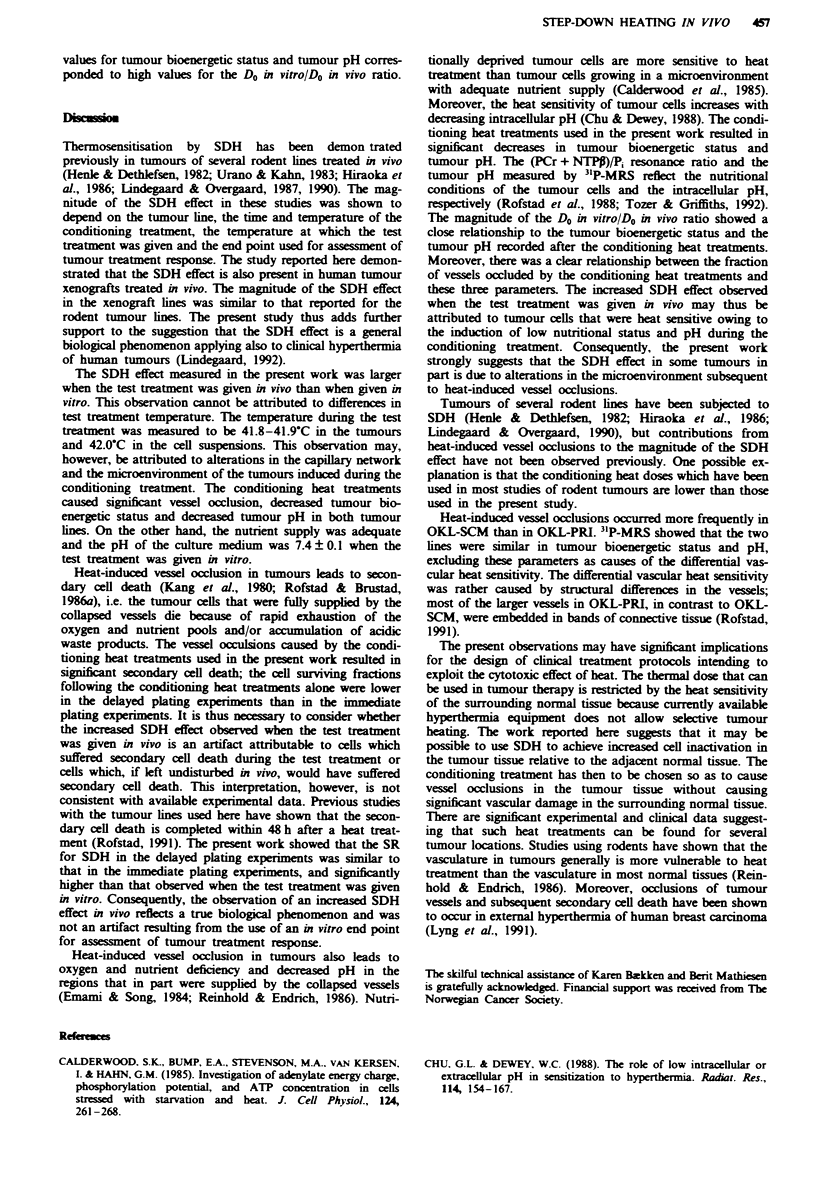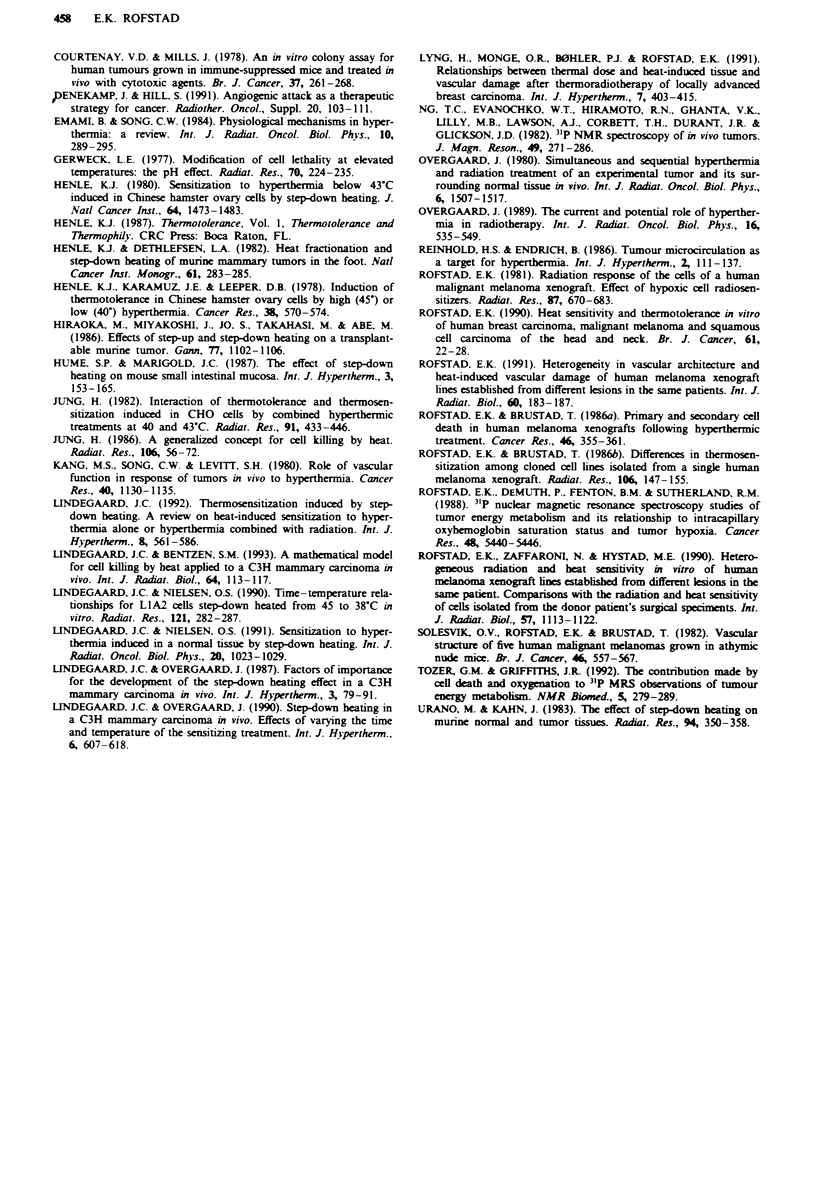# Step-down heating of human melanoma xenografts: effects of the tumour microenvironment.

**DOI:** 10.1038/bjc.1994.327

**Published:** 1994-09

**Authors:** E. K. Rofstad

**Affiliations:** Institute for Cancer Research, Norwegian Radium Hospital, Oslo.

## Abstract

Thermosensitisation by step-down heating (SDH) has previously been demonstrated in experimental rodent tumours. The purpose of the study reported here was to investigate whether the SDH effect in tumours in part may be attributed to heat-induced alterations in the capillary network and/or the microenvironment. Two human melanoma xenograft lines differing substantially in vascular parameters were selected for the study. A thermostatically regulated water bath was used for heat treatment. The conditioning treatment (44.5 degrees C or 45.5 degrees C for 15 min) was given in vivo, whereas the test treatment (42.0 degrees C for 45, 90, 135 or 180 min) was given either in vitro or in vivo. Treatment response was measured in vitro using a cell clonogenicity assay. Fraction of occluded vessels following heat treatment was assessed by examination of histological sections from tumours whose vascular network was filled with a contrast agent. Tumour bioenergetic status and tumour pH were measured by 31P magnetic resonance spectroscopy. The conditioning heat treatments caused significant vessel occlusion, decreased tumour bioenergetic status and decreased tumour pH in both tumour lines. The SDH effect measured when the test treatment was given in vivo was significantly increased relative to that measured when the test treatment was given in vitro. The magnitude of the increase showed a close relationship to fraction of occluded vessels, tumour bioenergetic status and tumour pH measured 90 min after treatment with 44.5 degrees C or 45.5 degrees C for 15 min. The increased SDH effect in vivo was probably attributable to tumour cells that were heat sensitive owing to the induction of low nutritional status and pH during the conditioning treatment. Consequently, the SDH effect in some tumours may in part be due to heat-induced alterations in the microenvironment. This suggests that SDH may be exploited clinically to achieve increased cell inactivation in tumours relative to the surrounding normal tissues.


Br. J. Cancer (1994), 70, 453-458 © Macmillan Press Ltd., 1994

### Step-down heating of human melanoma xenografts: effects of the tumour microenvironment

E.K. Rofstad

*Institute for Cancer Research, The Norwegian Radium Hospital, Montebello, 0310 Oslo, Norway.*

**Summary** Thermosensitisation by step-down heating (SDH) has previously been demonstrated in experimental rodent tumours. The purpose of the study reported here was to investigate whether the SDH effect in tumours in part may be attributed to heat-induced alterations in the capillary network and/or the microenvironment. Two human melanoma xenograft lines differing substantially in vascular parameters were selected for the study. A thermostatically regulated water bath was used for heat treatment. The conditioning treatment (44.5°C or 45.5°C for 15 min) was given *in vivo*, whereas the test treatment (42.0°C for 45, 90, 135 or 180 min) was given either *in vitro* or *in vivo*. Treatment response was measured *in vitro* using a cell clonogenicity assay. Fraction of occluded vessels following heat treatment was assessed by examination of histological sections from tumours whose vascular network was filled with a contrast agent. Tumour bioenergetic status and tumour pH were measured by $^{31}P$ magnetic resonance spectroscopy. The conditioning heat treatments caused significant vessel occlusion, decreased tumour bioenergetic status and decreased tumour pH in both tumour lines. The SDH effect measured when the test treatment was given *in vivo* was significantly increased relative to that measured when the test treatment was given *in vitro*. The magnitude of the increase showed a close relationship to fraction of occluded vessels, tumour bioenergetic status and tumour pH measured 90 min after treatment with 44.5°C or 45.5°C for 15 min. The increased SDH effect *in vivo* was probably attributable to tumour cells that were heat sensitive owing to the induction of low nutritional status and pH during the conditioning treatment. Consequently, the SDH effect in some tumours may in part be due to heat-induced alterations in the microenvironment. This suggests that SDH may be exploited clinically to achieve increased cell inactivation in tumours relative to the surrounding normal tissues.

The biological basis for the use of hyperthermia in the treatment of cancer is well established (Overgaard, 1989). The cell inactivation following hyperthermic treatment is known to be modulated by heat itself (Henle, 1987; Lindegaard, 1992). A short exposure to a high temperature may increase the cytotoxic effect of a subsequent heat treatment at a lower temperature. This phenomenon is termed thermosensitisation by step-down heating (SDH), and has been demonstrated in cells in culture (Henle *et al.*, 1978; Jung, 1982; Lindegaard & Nielsen, 1990), normal tissues (Henle & Dethlefsen, 1982; Hume & Marigold, 1987; Lindegaard & Nielsen, 1991) and experimental tumours (Henle & Dethlefsen, 1982; Hiraoka *et al.*, 1986; Lindegaard & Overgaard, 1990). The mechanisms governing the SDH effect are not known. However, it has been suggested that the SDH effect might be explained in terms of inhibition of repair of sublethal heat damage (Henle, 1980) or enhanced conversion of sublethal heat damage into lethal damage (Jung, 1986; Henle, 1987; Lindegaard & Bentzen, 1993). SDH could be of direct clinical importance, since even a short exposure to high temperature might increase the effect of an otherwise inadequate heat treatment (Lindegaard, 1992).

The cell inactivation in tumours following heat treatment is influenced substantially by the architecture of the microvasculature (Reinhold & Endrich, 1986) and the physiological conditions in the microenvironment of the cells prior to treatment (Emami & Song, 1984). An immature vascular network characterised by loss of hierarchy and tortuous capillaries with incomplete endothelial lining or interrupted basement membrane is more vulnerable to heat treatment than a mature vascular network with insignificant structural abnormalities (Reinhold & Endrich, 1986). Heat-induced vascular occlusions can lead to the development of massive tumour necrosis, a phenomenon frequently termed secondary cell death (Kang *et al.*, 1980; Rofstad & Brustad, 1986*a*; Denekamp & Hill, 1991). Tumour cells in a microenvironment characterised by low pH, oxygen deficiency and nutrient deprivation are more sensitive to heat treatment than tumour cells in a physiologically normal microenvironment (Gerweck, 1977; Overgaard, 1980). Moreover, the vascular and microenvironmental conditions in tumours can be modified extensively during a heat treatment, increasing the effectiveness of subsequent heat treatments (Emami & Song, 1984; Reinhold & Endrich, 1986).

The purpose of the work reported here was to investigate whether the SDH effect seen in tumours in part may be attributed to heat-induced alterations in the capillary network and/or the microenvironment. Two human melanoma xenograft lines being similar in cellular heat sensitivity (Rofstad *et al.*, 1990) but differing substantially in vascular heat sensitivity (Rofstad, 1991) were selected for the study.

### Materials and methods

### *Mice and tumour lines*

Male Balb/c *nu/nu* mice, 8-10 weeks old, were used. They were bred at the animal department of our institution and kept under specific-pathogen-free conditions at constant temperature (24-26°C) and humidity (30-50%). Sterilised food and tap water were given *ad libitum*.

Two melanoma xenograft lines established from the same patient, one from the primary tumour (OKL-PRI) and the other from a s.c. metastasis (OKL-SCM) (Rofstad *et al.*, 1990), were studied. The donor of the tumour tissue was a 34-year-old Caucasian female. The tumour lines were maintained in athymic mice by serial s.c. implantation of tumour fragments, approximately 2 × 2 × 2 mm. Tumours in passage 5 *in vivo* were grown s.c. in the left hind leg of athymic mice. The volume of the tumours ($V$) at the time of treatment, calculated as $V = \pi/6 \times a \times b^2$ ($a$ and $b$ are the longer and the shorter of two perpendicular diameters respectively), was within the range 200-300 $mm^3$. The vascular heat sensitivity of OKL-PRI and OKL-SCM is independent of tumour volume within this volume range (Rofstad, 1991). The distribution of tumour volumes was similar for OKL-PRI and OKL-SCM.

### *Heat treatment*

Heat treatment *in vivo* was given by immersing the tumour-bearing legs of non-anaesthetised mice into a thermostatically

Received 16 February 1994; and in revised form 11 May 1994.

regulated water bath (Rofstad & Brustad, 1986*a*). The mice were kept in specially designed jigs. The tumour-bearing legs were fixed to the jigs without impairing tumour blood supply, as verified in separate experiments by measuring the uptake of $^{86}$Rb. The temperature in the tumours, measured with a 0.3 mm needle thermocouple probe in separate groups of mice, was 0.1–0.2°C below the water bath temperature. The temperature equilibrium time was approximately 4 min (Rofstad & Burstad, 1986*a*). The body core temperature during treatment, measured with a rectal probe, was adjusted to 37–38°C by controlled air cooling of the mice.

Heat treatment *in vitro* was given by immersing plastic tubes containing single-cell suspensions into a thermostatically regulated water bath (Rofstad, 1990). The tubes were flushed with a gas mixture consisting of 5% carbon dioxide, 5% oxygen and 90% nitrogen and sealed prior to treatment. The pH of the culture medium during treatment was 7.4 ± 0.1, as verified in separate experiments by measuring pH at different times after flushing. Temperature equilibrium between the water bath and the culture medium was obtained within 4 min.

### *Cell survival assay*

Single-cell survival measured *in vitro* was used as the end point in all experiments. Tumours subjected to SDH *in vivo* were excised within 10 min (immediate plating experiments) or about 48 h (delayed plating experiments) after treatment. Single-cell suspensions were prepared from the tumours by mechanical disaggregation (Rofstad & Brustad, 1986*a*). The cell yield from untreated tumours was approximately $1 \times 10^8$ cells $g^{-1}$. The clonogenicity of the cells was assessed by using the Courtenay soft-agar assay (Courtenay & Mills, 1978). The soft agar was prepared from powdered agar (Bacto agar; Difco, Detroit, MI, USA), culture medium (Ham's F-12 medium containing 20% fetal calf serum, 250 mg $l^{-1}$ penicillin and 50 mg $l^{-1}$ streptomycin; Gibco-Biocult, Glasgow, UK) and erythrocytes from August rats (Rofstad, 1981). The tumour cells, embedded in aliquots of 1 ml of soft agar in tissue culture tubes (Falcon 2057 tubes; Becton-Dickinson, Oxnard, CA, USA), were incubated at 37°C for 4–5 weeks in an atmosphere of 5% carbon dioxide, 5% oxygen and 90% nitrogen for colony formation (Rofstad & Brustad, 1986*a*). Colonies containing more than 50 cells were counted by using a stereomicroscope (Rofstad, 1990). The plating efficiency of cells from untreated tumours was approximately 10% for both tumour lines. Cell surviving fractions were calculated from the cell yield and the plating efficiency as described by Rofstad and Brustad (1986*a*).

### *Assay for assessment of heat-induced vascular occlusions*

The vascular network of heated tumours was filled with a radio-opaque contrast medium administered via the abdominal aorta (Solesvik *et al.*, 1982). The contrast medium was composed of 100 ml 0.9% saline, 5 g of gelatin, 50 g of trilead tetroxide (red lead), 1 ml of detergent (Joy/Salo) and 5,000 units of heparin. Gelatin was dissolved in saline at 40°C. Lead was added in small doses under constant stirring. The solution was filtered and kept at 40°C while the detergent was added. Heparin was added immediately before use. The contrast medium was in liquid state at 40°C and coagulated at room temperature. It was injected (0.5 ml $min^{-1}$) at low and steady pressure to avoid damage to vascular structures. The viscous consistency of the contrast medium prevented small vessels from collapsing after the injection. It cannot be ruled out that the vascular dimensions were to some extent influenced by the contrast medium and the pressure used. However, repeated experiments showed that the method gave highly reproducible results.

The tumours were fixed in phosphate-buffered – 4% paraformaldehyde, dehydrated, embedded in paraffin casts and cut into 6-μm-thick sections. The sections were mounted on glass slides and stained with haematoxylin and eosin. Functional vessels appeared as dark circles or ellipses in the sections. Occluded vessels were unstained and showed a lumen that was densely packed with rigid, deformed erythrocytes. The fraction of occluded vessels was determined by differential counting according to stereological principles (Rofstad, 1991). The sections were examined at a magnification of 400 × by the use of a projecting light microscope and a counting frame, 20 × 20 cm (Solesvik *et al.*, 1982).

### *$^{31}$P magnetic resonance spectroscopy (MRS)*

$^{31}$P-MRS was performed in non-anaesthetised mice at a magnetic field strength of 34.6 MHz using solenoidal coils and a spectrometer with a horizontal magnet bore (Rofstad *et al.*, 1988). The homogeneity of the magnetic field was optimised for each individual tumour by shimming on the water proton resonance. The acquisition parameters were: 4K data points per free induction decay (FID); 4 μs pulse length; 1 kHz spectrum sweep width; 2,000 ms repetition time; 1,024 acquisitions per spectrum. The FIDs were subjected to an exponential line broadening of 10 Hz prior to Fourier transformation. Resonance areas were calculated from the best fits of Lorentzian lineshapes to phased, resolution-enhanced and baseline-corrected spectra. The (PCr + NTPβ)/$P_i$ resonance ratio was used as parameter for tumour bioenergetic status. Tumour pH was calculated from the chemical shift of the $P_i$ resonance with reference to the PCr resonance using the Henderson–Hasselbalch equation with $pK_a = 6.803$ (Ng *et al.*, 1982).

### *Statistical analysis*

The slope of a heat cell survival curve was expressed in terms of $D_0$, i.e. the heating time required to reduce the fraction of

**Table I** Heat survival curve parameters

| Heat treatment | OKL-PRI $D_0$ (min)[a] | OKL-PRI SR[a] | OKL-SCM $D_0$ (min)[a] | OKL-SCM SR[a] |
|---|---|---|---|---|
| Immediate plating[b] | | | | |
| 42.0°C (*t*) *in vivo* | 405 ± 42 | | 298 ± 27 | |
| 42.0°C (*t*) *in vivo* + 44.5°C (15 min) *in vivo* | 422 ± 46 | 1.0 ± 0.1 | 287 ± 31 | 1.0 ± 0.1 |
| 44.5°C (15 min) *in vivo* + 42.0°C (*t*) *in vitro* | 197 ± 20 | 2.1 ± 0.3 | 138 ± 15 | 2.2 ± 0.3 |
| 44.5°C (15 min) *in vivo* + 42.0°C (*t*) *in vivo* | 129 ± 14 | 3.1 ± 0.5 | 71 ± 8 | 4.2 ± 0.6 |
| 42.0°C (*t*) *in vivo* + 45.5°C (15 min) *in vivo* | 391 ± 40 | 1.0 ± 0.2 | 305 ± 33 | 1.0 ± 0.1 |
| 45.5°C (15 min) *in vivo* + 42.0°C (*t*) *in vitro* | 120 ± 16 | 3.4 ± 0.6 | 83 ± 12 | 3.6 ± 0.6 |
| 45.5°C (15 min) *in vivo* + 42.0°C (*t*) *in vivo* | 69 ± 11 | 5.9 ± 1.1 | 31 ± 5 | 9.6 ± 1.8 |
| Delayed plating[c] | | | | |
| 42.0°C (*t*) *in vivo* | 231 ± 22 | | 151 ± 17 | |
| 42.0°C (*t*) *in vivo* + 44.5°C (15 min) *in vivo* | 223 ± 34 | 1.0 ± 0.2 | 161 ± 28 | 0.9 ± 0.2 |
| 44.5°C (15 min) *in vivo* + 42.0°C (*t*) *in vivo* | 72 ± 14 | 3.2 ± 0.7 | 40 ± 7 | 3.8 ± 0.8 |

[a]Mean ± s.e. [b]The tumours were excised within 10 min after heat treatment for preparation of single-cell suspensions and plating *in vitro*. [c]The tumours were excised about 48 h after heat treatment for preparation of single-cell suspensions and plating *in vitro*.

surviving cells by a factor of $e^{-1}$. $D_0 \pm$ s.e. was determined by linear regression analysis. The Student *t*-test was used to test whether a biological parameter differed significantly between the two tumour lines. A significance level of $P = 0.05$ was used.

### Results

The SDH was performed by using 44.5°C (15 min) or 45.5°C (15 min) as conditioning treatment and 42.0°C (45, 90, 135 or 180 min) as test treatment, i.e. complete survival curves at 42.0°C were established for tumours pretreated with 44.5°C (15 min) or 45.5°C (15 min). The conditioning treatment was given *in vivo*, whereas the test treatment was given either *in vitro* or *in vivo*. The test treatment was always given 15 min after the conditioning treatment, allowing time for preparation of single-cell suspensions. The control experiments included single heating, i.e. 42.0°C (45, 90, 135 or 180 min), and step-up heating (SUH), i.e. 42.0°C (45, 90, 135 or 180 min) followed by 44.5°C (15 min) or 45.5°C (15 min). Two types of experiment were performed: immediate plating and delayed plating experiments, i.e. the tumours were excised within 10 min or about 48 h after treatment respectively (Table I).

The survival curves resulting from the immediate plating

**Figure 1** Heat survival curves at 42.0°C for the OKL-PRI (**a**) and the OKL-SCM (**b**) human melanoma xenograft lines. The following heat treatments were given: 42.0°C (*t*) *in vivo* (○), 42.0°C (*t*) *in vivo* + 44.5°C (15 min) *in vivo* (●), 44.5°C (15 min) *in vivo* + 42.0°C (*t*) *in vitro* (▽), 44.5°C (15 min) *in vivo* + 42.0°C (*t*) *in vivo* (▼), 42.0°C (*t*) *in vivo* + 45.5°C (15 min) *in vivo* (□), 45.5°C (15 min) *in vivo* + 42.0°C (*t*) *in vitro* (■) and 45.5°C (15 min) *in vivo* + 42.0°C (*t*) *in vivo* (△). The tumours were excised within 10 min after the treatment. The points and the vertical bars represent mean values and s.e.s, based on 5–8 independent measurements. Each of these measurements was based on the mean number of colonies in four tubes with cells from treated tumours and four tubes with cells from untreated control tumours.

**Figure 2** Heat survival curves at 42.0°C for the OKL-PRI (**a**) and the OKL-SCM (**b**) human melanoma xenograft lines. The following heat treatments were given: 42.0°C (*t*) *in vivo* (○), 42.0°C (*t*) *in vivo* + 44.5°C (15 min) *in vivo* (●) and 44.5°C (15 min) *in vivo* + 42.0°C (*t*) *in vivo* (▼). The tumours were excised about 48 h after the treatment. The points and the vertical bars represent mean values and s.e.s, based on 5–8 independent measurements. Each of these measurements was based on the mean number of colonies in four tubes with cells from treated tumours and four tubes with cells from untreated control tumours.

experiments are shown in Figure 1. $D_0$ and sensitisation ratio (SR), i.e. the $D_0$ for single heat treatment divided by the $D_0$ for SDH or SUH, are presented in Table I. The results were qualitatively similar for OKL-PRI and OKL-SCM. First, the $D_0$ for SUH was not significantly different from the $D_0$ for single heat treatment, i.e. the SR for SUH was not significantly different from 1.0. Second, the SR for SDH depended on the conditioning treatment and the experimental conditions during the test treatment, but was significantly higher than 1.0 ($P<0.05$). Thus, a conditioning treatment of 45.5°C (15 min) resulted in a significantly higher SR than a conditioning treatment of 44.5°C (15 min) ($P<0.05$). Moreover, the SR was significantly higher when the test treatment was given *in vivo* than when given *in vitro* ($P<0.05$). The $D_0$ for single heat treatment at 42.0°C *in vitro* was 385 ± 40 min for OKL-PRI and 312 ± 35 for OKL-SCM (data not shown), i.e. indistinguishable from the $D_0$ for single heat treatment at 42.0°C *in vivo*. On the other hand, the results were quantitatively different for OKL-PRI and OKL-SCM. Thus, the $D_0$ was significantly higher for OKL-PRI than for OKL-SCM ($P<0.05$) and the SR for SDH was significantly higher for OKL-SCM than for OKL-PRI when the test treatment was given *in vivo* ($P<0.05$). However, OKL-PRI and OKL-SCM showed similar SR for SDH when the test treatment was given *in vitro*.

The survival curves resulting from the delayed plating experiments are shown in Figure 2. These curves were qualitatively similar to those presented in Figure 1. However, delayed plating resulted in reduced surviving fraction following the conditioning treatment alone ($P<0.05$) and a decrease in the $D_0$ ($P<0.05$) for both OKL-PRI and OKL-SCM. The SR for SDH *in vivo* in the delayed plating experiments was similar to the SR for SDH *in vivo* in the immediate plating experiments, but significantly higher than the SR for SDH when the test treatment was given *in vitro* in the immediate plating experiments (Table I). The rapid decay of the SDH effect (Rofstad & Brustad, 1986*b*) prevented the design of rational delayed plating experiments with the test treatment given *in vitro*.

The SDH effect measured when the test treatment was given *in vivo* was increased relative to that measured when the test treatment was given *in vitro* (Figure 1). The magnitude of the increase, i.e. the $D_0$ for SDH measured when the test treatment was given *in vitro* divided by the $D_0$ for SDH measured when the test treatment was given *in vivo* ($D_0$ *in vitro*/$D_0$ *in vivo*), is illustrated in Figure 3a. The conditioning heat treatments caused significant vessel occlusion. The fraction of occluded vessels 90 min after treatment with 44.5°C (15 min) or 45.5°C (15 min) is shown in Figure 3b. Fraction of occluded vessels and the magnitude of the $D_0$ *in vitro*/$D_0$ *in vivo* ratio paralleled one another; the sequence from high to low values of these two parameters was: OKL-SCM (45.5°C, 15 min); OKL-SCM (44.5°C, 15 min); OKL-PRI (45.5°C, 15 min); OKL-PRI (44.5°C, 15 min). However, the s.e.s of the $D_0$ *in vitro*/$D_0$ *in vivo* ratios were overlapping.

Moreover, the conditioning heat treatments caused significant lowering of tumour bioenergetic status (Figure 4a) and tumour pH (Figure 4b). Figure 4 is based on $^{31}$P-MRS measurements performed 90 min after treatment with 44.5°C (15 min) or 45.5°C (15 min). The decrease was significantly larger for OKL-SCM than for OKL-PRI ($P<0.05$) and significantly larger for 45.5°C (15 min) than for 44.5°C (15 min) ($P<0.05$) for both tumour bioenergetic status and tumour pH. The absolute values for tumour bioenergetic status and tumour pH after heat treatment showed a close relationship to the $D_0$ *in vitro*/$D_0$ *in vivo* ratio (Figure 3a); low

**Figure 3** $D_0$ *in vitro*/$D_0$ *in vivo* ratio, i.e. the $D_0$ for SDH measured when the test treatment was given at 42.0°C *in vitro* divided by the $D_0$ for SDH measured when the test treatment was given at 42.0°C *in vivo* (**a**) and fraction of occluded vessels measured 90 min after completion of the conditioning treatment (**b**) for the OKL-PRI and the OKL-SCM human melanoma xenograft lines. The conditioning treatment was 44.5°C (15 min) ( ▨ ) or 45.5°C (15 min) ( □ ). The columns and the vertical bars represent mean values and s.e.s, based on the heat survival curve parameters in Table I (**a**) and 6–10 individual tumours (**b**).

**Figure 4** Tumour bioenergetic status, i.e. the (PCr + NTPβ)/$P_i$ resonance ratio (**a**) and tumour pH (**b**) measured by $^{31}$P-MRS for the OKL-PRI and the OKL-SCM human melanoma xenograft lines. $^{31}$P-MRS was performed prior to treatment ( ■ ), 90 min after treatment with 44.5°C (15 min) ( ▩ ) or 90 min after treatment with 45.5°C (15 min) ( □ ). The columns and the vertical bars represent mean values and s.e.s, based on 8–10 individual tumours.

values for tumour bioenergetic status and tumour pH corresponded to high values for the $D_0$ *in vitro*/$D_0$ *in vivo* ratio.

### Discussion

Thermosensitisation by SDH has been demon trated previously in tumours of several rodent lines treated *in vivo* (Henle & Dethlefsen, 1982; Urano & Kahn, 1983; Hiraoka *et al.*, 1986; Lindegaard & Overgaard, 1987, 1990). The magnitude of the SDH effect in these studies was shown to depend on the tumour line, the time and temperature of the conditioning treatment, the temperature at which the test treatment was given and the end point used for assessment of tumour treatment response. The study reported here demonstrated that the SDH effect is also present in human tumour xenografts treated *in vivo*. The magnitude of the SDH effect in the xenograft lines was similar to that reported for the rodent tumour lines. The present study thus adds further support to the suggestion that the SDH effect is a general biological phenomenon applying also to clinical hyperthermia of human tumours (Lindegaard, 1992).

The SDH effect measured in the present work was larger when the test treatment was given *in vivo* than when given *in vitro*. This observation cannot be attributed to differences in test treatment temperature. The temperature during the test treatment was measured to be 41.8–41.9°C in the tumours and 42.0°C in the cell suspensions. This observation may, however, be attributed to alterations in the capillary network and the microenvironment of the tumours induced during the conditioning treatment. The conditioning heat treatments caused significant vessel occlusion, decreased tumour bioenergetic status and decreased tumour pH in both tumour lines. On the other hand, the nutrient supply was adequate and the pH of the culture medium was $7.4 \pm 0.1$ when the test treatment was given *in vitro*.

Heat-induced vessel occlusion in tumours leads to secondary cell death (Kang *et al.*, 1980; Rofstad & Brustad, 1986*a*), i.e. the tumour cells that were fully supplied by the collapsed vessels die because of rapid exhaustion of the oxygen and nutrient pools and/or accumulation of acidic waste products. The vessel occulsions caused by the conditioning heat treatments used in the present work resulted in significant secondary cell death; the cell surviving fractions following the conditioning heat treatments alone were lower in the delayed plating experiments than in the immediate plating experiments. It is thus necessary to consider whether the increased SDH effect observed when the test treatment was given *in vivo* is an artifact attributable to cells which suffered secondary cell death during the test treatment or cells which, if left undisturbed *in vivo*, would have suffered secondary cell death. This interpretation, however, is not consistent with available experimental data. Previous studies with the tumour lines used here have shown that the secondary cell death is completed within 48 h after a heat treatment (Rofstad, 1991). The present work showed that the SR for SDH in the delayed plating experiments was similar to that in the immediate plating experiments, and significantly higher than that observed when the test treatment was given *in vitro*. Consequently, the observation of an increased SDH effect *in vivo* reflects a true biological phenomenon and was not an artifact resulting from the use of an *in vitro* end point for assessment of tumour treatment response.

Heat-induced vessel occlusion in tumours also leads to oxygen and nutrient deficiency and decreased pH in the regions that in part were supplied by the collapsed vessels (Emami & Song, 1984; Reinhold & Endrich, 1986). Nutritionally deprived tumour cells are more sensitive to heat treatment than tumour cells growing in a microenvironment with adequate nutrient supply (Calderwood *et al.*, 1985). Moreover, the heat sensitivity of tumour cells increases with decreasing intracellular pH (Chu & Dewey, 1988). The conditioning heat treatments used in the present work resulted in significant decreases in tumour bioenergetic status and tumour pH. The (PCr + NTPβ)/$P_i$ resonance ratio and the tumour pH measured by $^{31}$P-MRS reflect the nutritional conditions of the tumour cells and the intracellular pH, respectively (Rofstad *et al.*, 1988; Tozer & Griffiths, 1992). The magnitude of the $D_0$ *in vitro*/$D_0$ *in vivo* ratio showed a close relationship to the tumour bioenergetic status and the tumour pH recorded after the conditioning heat treatments. Moreover, there was a clear relationship between the fraction of vessels occluded by the conditioning heat treatments and these three parameters. The increased SDH effect observed when the test treatment was given *in vivo* may thus be attributed to tumour cells that were heat sensitive owing to the induction of low nutritional status and pH during the conditioning treatment. Consequently, the present work strongly suggests that the SDH effect in some tumours in part is due to alterations in the microenvironment subsequent to heat-induced vessel occlusions.

Tumours of several rodent lines have been subjected to SDH (Henle & Dethlefsen, 1982; Hiraoka *et al.*, 1986; Lindegaard & Overgaard, 1990), but contributions from heat-induced vessel occlusions to the magnitude of the SDH effect have not been observed previously. One possible explanation is that the conditioning heat doses which have been used in most studies of rodent tumours are lower than those used in the present study.

Heat-induced vessel occlusions occurred more frequently in OKL-SCM than in OKL-PRI. $^{31}$P-MRS showed that the two lines were similar in tumour bioenergetic status and pH, excluding these parameters as causes of the differential vascular heat sensitivity. The differential vascular heat sensitivity was rather caused by structural differences in the vessels; most of the larger vessels in OKL-PRI, in contrast to OKL-SCM, were embedded in bands of connective tissue (Rofstad, 1991).

The present observations may have significant implications for the design of clinical treatment protocols intending to exploit the cytotoxic effect of heat. The thermal dose that can be used in tumour therapy is restricted by the heat sensitivity of the surrounding normal tissue because currently available hyperthermia equipment does not allow selective tumour heating. The work reported here suggests that it may be possible to use SDH to achieve increased cell inactivation in the tumour tissue relative to the adjacent normal tissue. The conditioning treatment has then to be chosen so as to cause vessel occlusions in the tumour tissue without causing significant vascular damage in the surrounding normal tissue. There are significant experimental and clinical data suggesting that such heat treatments can be found for several tumour locations. Studies using rodents have shown that the vasculature in tumours generally is more vulnerable to heat treatment than the vasculature in most normal tissues (Reinhold & Endrich, 1986). Moreover, occlusions of tumour vessels and subsequent secondary cell death have been shown to occur in external hyperthermia of human breast carcinoma (Lyng *et al.*, 1991).

The skilful technical assistance of Karen Bækken and Berit Mathiesen is gratefully acknowledged. Financial support was received from The Norwegian Cancer Society.

### References

CALDERWOOD, S.K., BUMP, E.A., STEVENSON, M.A., VAN KERSEN, I. & HAHN, G.M. (1985). Investigation of adenylate energy charge, phosphorylation potential, and ATP concentration in cells stressed with starvation and heat. *J. Cell Physiol.*, **124**, 261–268.

CHU, G.L. & DEWEY, W.C. (1988). The role of low intracellular or extracellular pH in sensitization to hyperthermia. *Radiat. Res.*, **114**, 154–167.

COURTENAY, V.D. & MILLS, J. (1978). An *in vitro* colony assay for human tumours grown in immune-suppressed mice and treated *in vivo* with cytotoxic agents. *Br. J. Cancer*, **37**, 261-268.

DENEKAMP, J. & HILL, S. (1991). Angiogenic attack as a therapeutic strategy for cancer. *Radiother. Oncol.*, Suppl. 20, 103-111.

EMAMI, B. & SONG, C.W. (1984). Physiological mechanisms in hyperthermia: a review. *Int. J. Radiat. Oncol. Biol. Phys.*, **10**, 289-295.

GERWECK, L.E. (1977). Modification of cell lethality at elevated temperatures: the pH effect. *Radiat. Res.*, **70**, 224-235.

HENLE, K.J. (1980). Sensitization to hyperthermia below 43°C induced in Chinese hamster ovary cells by step-down heating. *J. Natl Cancer Inst.*, **64**, 1473-1483.

HENLE, K.J. (1987). *Thermotolerance*, Vol. 1, *Thermotolerance and Thermophily*. CRC Press: Boca Raton, FL.

HENLE, K.J. & DETHLEFSEN, L.A. (1982). Heat fractionation and step-down heating of murine mammary tumors in the foot. *Natl Cancer Inst. Monogr.*, **61**, 283-285.

HENLE, K.J., KARAMUZ, J.E. & LEEPER, D.B. (1978). Induction of thermotolerance in Chinese hamster ovary cells by high (45°) or low (40°) hyperthermia. *Cancer Res.*, **38**, 570-574.

HIRAOKA, M., MIYAKOSHI, J., JO, S., TAKAHASI, M. & ABE, M. (1986). Effects of step-up and step-down heating on a transplantable murine tumor. *Gann*, **77**, 1102-1106.

HUME, S.P. & MARIGOLD, J.C. (1987). The effect of step-down heating on mouse small intestinal mucosa. *Int. J. Hypertherm.*, **3**, 153-165.

JUNG, H. (1982). Interaction of thermotolerance and thermosensitization induced in CHO cells by combined hyperthermic treatments at 40 and 43°C. *Radiat. Res.*, **91**, 433-446.

JUNG, H. (1986). A generalized concept for cell killing by heat. *Radiat. Res.*, **106**, 56-72.

KANG, M.S., SONG, C.W. & LEVITT, S.H. (1980). Role of vascular function in response of tumors *in vivo* to hyperthermia. *Cancer Res.*, **40**, 1130-1135.

LINDEGAARD, J.C. (1992). Thermosensitization induced by step-down heating. A review on heat-induced sensitization to hyperthermia alone or hyperthermia combined with radiation. *Int. J. Hypertherm.*, **8**, 561-586.

LINDEGAARD, J.C. & BENTZEN, S.M. (1993). A mathematical model for cell killing by heat applied to a C3H mammary carcinoma *in vivo*. *Int. J. Radiat. Biol.*, **64**, 113-117.

LINDEGAARD, J.C. & NIELSEN, O.S. (1990). Time-temperature relationships for L1A2 cells step-down heated from 45 to 38°C *in vitro*. *Radiat. Res.*, **121**, 282-287.

LINDEGAARD, J.C. & NIELSEN, O.S. (1991). Sensitization to hyperthermia induced in a normal tissue by step-down heating. *Int. J. Radiat. Oncol. Biol. Phys.*, **20**, 1023-1029.

LINDEGAARD, J.C. & OVERGAARD, J. (1987). Factors of importance for the development of the step-down heating effect in a C3H mammary carcinoma *in vivo*. *Int. J. Hypertherm.*, **3**, 79-91.

LINDEGAARD, J.C. & OVERGAARD, J. (1990). Step-down heating in a C3H mammary carcinoma *in vivo*. Effects of varying the time and temperature of the sensitizing treatment. *Int. J. Hypertherm.*, **6**, 607-618.

LYNG, H., MONGE, O.R., BØHLER, P.J. & ROFSTAD, E.K. (1991). Relationships between thermal dose and heat-induced tissue and vascular damage after thermoradiotherapy of locally advanced breast carcinoma. *Int. J. Hypertherm.*, **7**, 403-415.

NG, T.C., EVANOCHKO, W.T., HIRAMOTO, R.N., GHANTA, V.K., LILLY, M.B., LAWSON, A.J., CORBETT, T.H., DURANT, J.R. & GLICKSON, J.D. (1982). $^{31}$P NMR spectroscopy of *in vivo* tumors. *J. Magn. Reson.*, **49**, 271-286.

OVERGAARD, J. (1980). Simultaneous and sequential hyperthermia and radiation treatment of an experimental tumor and its surrounding normal tissue *in vivo*. *Int. J. Radiat. Oncol. Biol. Phys.*, **6**, 1507-1517.

OVERGAARD, J. (1989). The current and potential role of hyperthermia in radiotherapy. *Int. J. Radiat. Oncol. Biol. Phys.*, **16**, 535-549.

REINHOLD, H.S. & ENDRICH, B. (1986). Tumour microcirculation as a target for hyperthermia. *Int. J. Hypertherm.*, **2**, 111-137.

ROFSTAD, E.K. (1981). Radiation response of the cells of a human malignant melanoma xenograft. Effect of hypoxic cell radiosensitizers. *Radiat. Res.*, **87**, 670-683.

ROFSTAD, E.K. (1990). Heat sensitivity and thermotolerance *in vitro* of human breast carcinoma, malignant melanoma and squamous cell carcinoma of the head and neck. *Br. J. Cancer*, **61**, 22-28.

ROFSTAD, E.K. (1991). Heterogeneity in vascular architecture and heat-induced vascular damage of human melanoma xenograft lines established from different lesions in the same patients. *Int. J. Radiat. Biol.*, **60**, 183-187.

ROFSTAD, E.K. & BRUSTAD, T. (1986*a*). Primary and secondary cell death in human melanoma xenografts following hyperthermic treatment. *Cancer Res.*, **46**, 355-361.

ROFSTAD, E.K. & BRUSTAD, T. (1986*b*). Differences in thermosensitization among cloned cell lines isolated from a single human melanoma xenograft. *Radiat. Res.*, **106**, 147-155.

ROFSTAD, E.K., DEMUTH, P., FENTON, B.M. & SUTHERLAND, R.M. (1988). $^{31}$P nuclear magnetic resonance spectroscopy studies of tumor energy metabolism and its relationship to intracapillary oxyhemoglobin saturation status and tumor hypoxia. *Cancer Res.*, **48**, 5440-5446.

ROFSTAD, E.K., ZAFFARONI, N. & HYSTAD, M.E. (1990). Heterogeneous radiation and heat sensitivity *in vitro* of human melanoma xenograft lines established from different lesions in the same patient. Comparisons with the radiation and heat sensitivity of cells isolated from the donor patient's surgical speciments. *Int. J. Radiat. Biol.*, **57**, 1113-1122.

SOLESVIK, O.V., ROFSTAD, E.K. & BRUSTAD, T. (1982). Vascular structure of five human malignant melanomas grown in athymic nude mice. *Br. J. Cancer*, **46**, 557-567.

TOZER, G.M. & GRIFFITHS, J.R. (1992). The contribution made by cell death and oxygenation to $^{31}$P MRS observations of tumour energy metabolism. *NMR Biomed.*, **5**, 279-289.

URANO, M. & KAHN, J. (1983). The effect of step-down heating on murine normal and tumor tissues. *Radiat. Res.*, **94**, 350-358.